# Strain-Induced Frequency Splitting in PT Symmetric Coupled Silicon Resonators

**DOI:** 10.3390/mi15101278

**Published:** 2024-10-21

**Authors:** Lifeng Wang, Shangyang Zhang, Qunce Yuan

**Affiliations:** Key Laboratory of MEMS of the Ministry of Education, School of Electronic Science & Engineering, Southeast University, Nanjing 210096, China; 230228378@seu.edu.cn (S.Z.); ym151221@163.com (Q.Y.)

**Keywords:** PT symmetry, coupled resonators, silicon resonators, strain-induced, frequency splitting

## Abstract

When two resonators of coupled silicon resonators are identical and the gain on one side is equal to the loss on the other side, a parity-time (PT) symmetric-coupled silicon resonator is formed. As non-Hermitian systems, the PT-symmetric systems have exhibited many special properties and interesting phenomena. This paper proposes the strain-induced frequency splitting in PT symmetry-coupled silicon resonators. The frequency splitting of the PT system caused by strain perturbations is derived and simulated. Theory and simulation both indicate that the PT system is more sensitive to strain perturbation near the exceptional point (EP) point. Then, a feedback circuit is designed to achieve the negative damping required for PT symmetry. Based on a simple silicon-on-insulator (SOI) process, the silicon resonator chip is successfully fabricated. After that, the PT-symmetric-coupled silicon resonators are successfully constructed, and the frequency splitting phenomenon caused by strain is observed experimentally.

## 1. Introduction

In 1998, Professor C. M. Bender of University of Washington proposed a parity-time (PT)-symmetric Hamiltonian. This PT-symmetric Hamiltonian does not have Hermitian properties, but still has a real eigenvalue spectrum [1]. Here, P and T represent parity transformation and time transformation, respectively. According to Professor Bender’s theory, the condition for Hamiltonian H to have PT symmetry is that the inner product of the PT transformation operator and the H operator is 0, denoted as [PT, H] = 0. It can also be understood as the condition that a system’s Hamiltonian remains unchanged after undergoing parity transformation (coordinate x transformation to −x) and time transformation (time t transformation to −t). After Professor Bender proposed the concept of PT symmetry, many researchers quickly joined the study on this non-Hermitian PT symmetry system. So far, the theoretical framework of PT-symmetric non-Hermitian quantum systems has been established [2,3].

Compared to Hermitian symmetry in mathematical form, PT symmetry is closer to physical symmetry. Therefore, in addition to research in quantum fields, PT symmetry theory has also been validated in different types of classical physical systems. In 2007, R. El Ganainy et al. from the University of Central Florida first proposed an optical PT-symmetric structure and formulated a coupled-mode theory (CMT) appropriate for PT-symmetric optical elements [4]. In 2008, Technion-Israel Institute of Technology’s S Klaiman et al. first observed the phase transition process of PT-symmetric optical structures from exact PT symmetry to broken PT symmetry and identified the PT symmetry exceptional point (EP) [5]. In 2011, J. Schindler from Wesleyan University first proposed a PT-symmetric electrical system based on a coupled LCR resonant circuit. The theoretical model for the electrical PT system was established and experimentally verified [6]. In 2014, X. Zhu et al. from the University of California introduced the concept of acoustic PT symmetry. Combining this acoustic PT-symmetric medium with transformation acoustics, two-dimensional symmetric acoustic cloaks that were unidirectionally invisible in a prescribed direction were designed [7]. In 2016, R. Fleury et al. from the University of Texas systematically expounded the theoretical model and implementation method of acoustic PT-symmetric system [8].

Gradually, some special properties and interesting phenomena of PT symmetry systems have been discovered [9,10,11,12,13,14]. Z. Lin et al. from Wesleyan University found that PT-symmetric optical periodic structures exhibit unidirectional invisibility properties. Near the PT breaking point, if the light wave enters from the left side of the PT structure, it will exit from the right side completely unaffected; and if the light wave enters from the right side, its reflected wave will be enhanced [9]. Wesleyan University’s H. Ramezani et al. discovered the Talbot effect in PT-symmetric optical systems. When the input field pattern respects specific periodicities, the complex PT-symmetric photonic lattices can lead to a new class of self-imaging Talbot effects [10]. University of Central Florida’s H. Hodaei et al. developed a micro-resonant ring laser using the PT symmetry principle. By harnessing notions from PT symmetry, stable single-longitudinal mode operation can be readily achieved in a system of coupled micro-ring lasers. The selective breaking of PT symmetry can be used to systematically enhance the maximum attainable output power in the desired mode [12]. S. Assawaworrarit et al. from Stanford University proposed theoretically and demonstrated experimentally that a parity-time symmetric circuit incorporating a nonlinear gain saturation element provides robust wireless power transfer. The transfer efficiency can remain near unity over a distance variation of approximately one meter, without the need for any tuning [14]. Wayne State University’s P Chen et al. proposed the PTX symmetric theory and significantly improved the readout signal strength of LC sensors using this theory [15]. B. Zhou et al. from Southeast University constructed a PT-symmetric LC sensing system working in the broken PT regime. Experiments showed that its wireless readout distance was nearly four times longer than traditional methods [16]. Recently, a type of artificial material called metamaterial has become a research hotspot due to its unusual electromagnetic properties [17,18]. P. R. Charumathi et al. revealed that the PT metamaterials exhibit several unusual light–matter interactions that could be exploited for realizing novel applications [19].

As a special point in PT phase transition, the EP point exhibits some outstanding characteristics. Z Liu et al. from Tsinghua University showed that it is possible to realize ultrasensitive optomechanical PT-symmetric microcavities whose sensitivity is at least two orders of magnitude better than single-cavity optomechanical transducers [20]; Tongji University’s C Zeng et al. reported the study of high-order EP in PT-symmetric electrical systems, and found that the sensitivity of the system was enhanced by nearly 10 times when the perturbation frequency was less than 0.4 kHz [21]. H. Hodaei et al. from the University of Central Florida found that the higher the order of EP in PT-symmetric micro-resonant rings, the higher the sensitivity of their perturbation response [22]. Z. Dong et al. from the National University of Singapore used an EP-locked PT-symmetric circuit to increase the sensitivity of implantable passive wireless sensors by 3.2 times [23]. The University of Texas at Austin’s Z Xiao et al. designed PT-symmetric circuits with a sixth order EP, which can be used for readout of capacitive sensors and has the advantages of high sensitivity, high resolution, and low thermal noise [24].

Our team successfully constructed PT systems based on coupled LC resonators and coupled silicon resonators, and established corresponding theoretical models [16,25,26]. Based on our previous work, this paper studies the strain-induced frequency splitting in PT symmetry-coupled silicon resonators. Theoretical derivation, simulation analysis and experimental measurements will be conducted to study the strain perturbation on the PT system. Due to the particularity of the EP, our observation will start from the EP point of the PT system. The main content of this paper is organized as follows: Section 2 derives the theory of PT symmetry enhanced strain sensing; Section 3 designs the structure of the PT-symmetric-coupled silicon resonators and simulates its mechanical and circuit model; Section 4 shows the fabrication process of the silicon resonators, and gives the experimental results; finally, Section 5 makes some conclusions.

## 2. Principle

**Figure 1:**
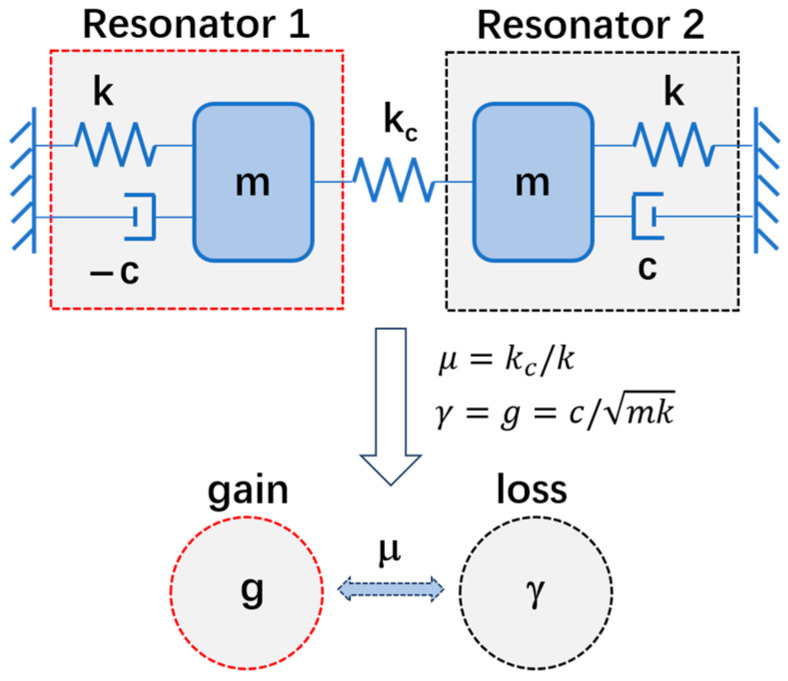
Schematic of the PT-symmetric-coupled silicon resonators.

Figure 1 shows the schematic of the PT-symmetric-coupled silicon resonators. Resonator 1 and Resonator 2 have the same mass *m* and elastic coefficient *k*, and the coupling coefficient between the two resonators is *k_c_*. Different from the traditional coupled silicon resonators which includes two loss resonators [27,28], the proposed coupled silicon resonators here consist of one loss resonator and one gain resonator. When the positive damping of Resonator 2 and the negative damping of Resonator 1 are equal in magnitude, the two resonators form a PT-symmetric system.

According to Newton’s law, the equations of the motions of the coupled silicon resonators are given as:

(1a) $$m\frac{d^{2}x_{1}}{dt^{2}}-c\frac{dx_{1}}{dt}+kx_{1}+k_{c}\left(x_{1}-x_{2}\right)=0$$

(1b) $$m\frac{d^{2}x_{2}}{dt^{2}}+c\frac{dx_{1}}{dt}+kx_{2}+k_{c}(x_{2}-x_{1})=0$$

in which *c* is the damping coefficient, and *x_n_* (*n* = 1,2) denotes the vibration displacements of the two resonators, respectively. In Equation (1), swapping the indices of *x* (P reverse) and changing the sign of *t* (T reverse) leaves the equations unchanged, which conforms to the definition of PT symmetry. The gain of Resonator 1 and the loss of Resonator 2 can be defined as $g=\gamma =c/\sqrt{mk}$, the coupling strength between the two resonators can be defined as $\mu =k_{c}/k$, then the PT symmetry coupled silicon resonators can be simplified as a PT-symmetric dimer, as shown in Figure 1.

Taking $x_{n}\left(t\right)\propto x_{n}e^{i\omega t}$, Equation (1) can be rewritten as:

(2) $$\left[\begin{array}{cc} 1+\mu -i\omega g-\omega^{2} & -\mu \\ -\mu & 1+\mu +i\omega \gamma -\omega^{2} \end{array}\right]\left[\begin{array}{c} x_{1}\\ x_{2} \end{array}\right]=0$$

where ω is scaled by $\omega_{0}=\sqrt{k/m}$, which is the resonant frequency of the single silicon resonator.

Here, the coupled silicon resonators are set in a weakly coupled state (μ≪1). When the system is in weak coupling, Equation (2) can be simplified to:

(3) $$\left[\begin{array}{cc} 1+\frac{\mu -ig}{2}-\omega & -\frac{\mu }{2}\\ -\frac{\mu }{2} & 1+\frac{\mu +i\gamma }{2}-\omega \end{array}\right]\left[\begin{array}{c} x_{1}\\ x_{2} \end{array}\right]=0$$

Solving Equation (3), the eigenfrequencies of the PT system can be obtained as:

(4) $$\omega_{\pm }=1+\frac{\mu }{2}\pm \frac{1}{2}\sqrt{\mu^{2}-\gamma^{2}}$$

In Equation (4), when *μ* > *γ*, the system is in an exact PT-symmetric phase. The system has two real eigenfrequencies and the frequency splitting Δ*w* is:

(5) $$∆\omega =\sqrt{\mu^{2}-\gamma^{2}}$$

In Equation (4), when *μ* < *γ*, the system is in a broken PT-symmetric phase. The eigenfrequencies have imaginary parts, and the system has only one real eigenfrequency; between these two regimes, when *μ* = *γ*, the system is in an EP, where the transition of the two different phases of PT symmetry occurs.

According to the above analysis, the eigenfrequencies merge in the broken PT symmetry regime. In another word, the frequency splitting Δ*w* of the PT system remains zero at this regime. Therefore, the strain-induced frequency splitting only occurs in the exact PT symmetry regime starting from EP.

When strain is applied to the coupled silicon resonators, both resonators are simultaneously subjected to elastic coefficient perturbations *δ*. After perturbation, the parameters of the coupled resonator are rewritten as:

(6a) $$\mu^{\prime }=\frac{\mu }{1-\delta }$$

(6b) $$\gamma^{\prime }=\frac{\gamma }{\sqrt{1-\delta }}$$

(6c) $$\omega_{0}^{\prime }=\sqrt{1-\delta }\omega_{0}$$

Substituting Equation (6) into Equation (3), and setting the initial state of the PT system at EP (*μ* = *γ*), Equation (5) can be rewritten as:

(7) $$∆\omega^{\prime }=\sqrt{{\mu^{\prime }}^{2}-{\gamma^{\prime }}^{2}}=\frac{\sqrt{\delta }}{1-\delta }\mu$$

Normalizing Δ*w’* by *w*_0_, the frequency splitting induced by strain can be obtained as:

(8) $$∆\omega =\frac{\sqrt{\delta }}{\sqrt{1-\delta }}\mu$$

## 3. Design and Simulation

### 3.1. Structural Design

**Figure 2:**
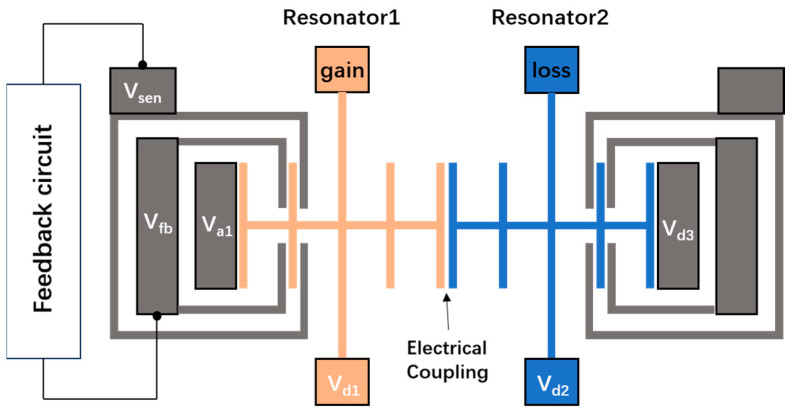
The structural design of the PT-symmetric-coupled silicon resonators.

Figure 2 shows the structural design of the PT-symmetric-coupled silicon resonators according to Figure 1. Resonator 1 and Resonator 2 are structurally symmetrical, while Resonator 1 is a gain resonator and Resonator 2 is a loss resonator. To create a gain effect on Resonator 1, the electrode V_sen_ detects the vibration of Resonator 1 and applies back to the V_fb_ electrode through a feedback circuit loop. The magnitude of gain can be adjusted by the feedback circuit. The loss of Resonator 2 is determined by its own parameters as well as the ambient air pressure, and its magnitude can be adjusted by the air pressure. The two resonators are coupled through electrical coupling. The coupling strength can be adjusted by the DC voltages V_d1_ and V_d2_. AC voltage V_a1_ initiates the motion of the resonator. The DC voltage V_d3_ is used to eliminate the asymmetric effects caused by fabrication deviations.

### 3.2. Mechanical Model Simulation

**Figure 3:**
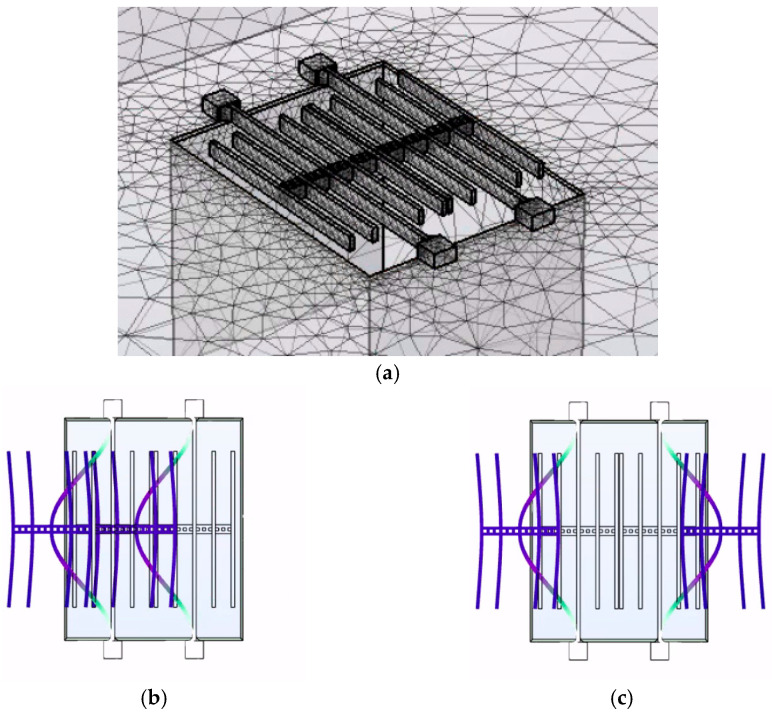
FEM simulation of the PT-symmetric-coupled silicon resonators. (**a**) simulation model; (**b**) the in-phase mode of vibration and (**c**) out-of-phase mode of vibration.

The finite element simulation software COMSOL Multiphysics 6.2 was used to simulate and analyze the PT-symmetric-coupled silicon resonators, as shown in Figure 3a. The silicon resonators were fabricated based on silicon-on-insulator (SOI) process. The thickness of the silicon beam was 25 μm, the thickness of the substrate was 400 μm, and the gap between the silicon beam and the substrate was 2 μm. The size of silicon resonators is shown in Table 1.

Figure 3b,c show the simulated two basic vibration modes of the coupled resonators, the in-phase mode (first mode) and the out-of-phase mode (second mode). The in-phase mode corresponds to the lower eigenfrequency of the coupled resonator system, while the out-of-phase mode corresponds to a higher eigenfrequency of the coupled resonator system.

**Figure 4:**
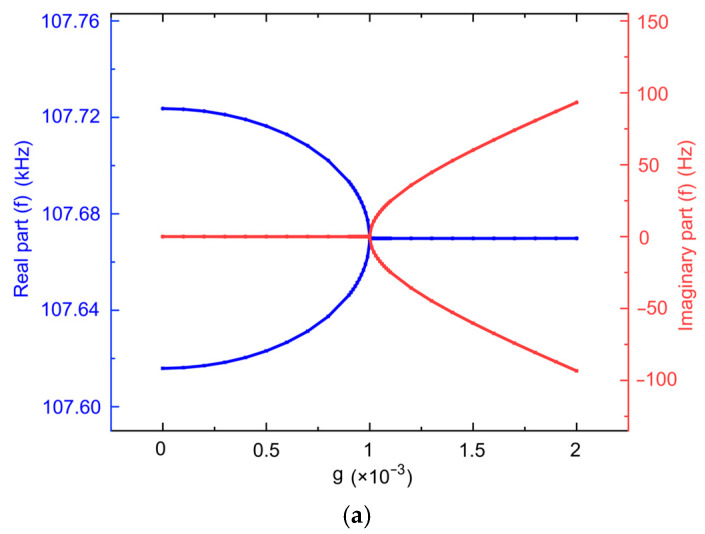

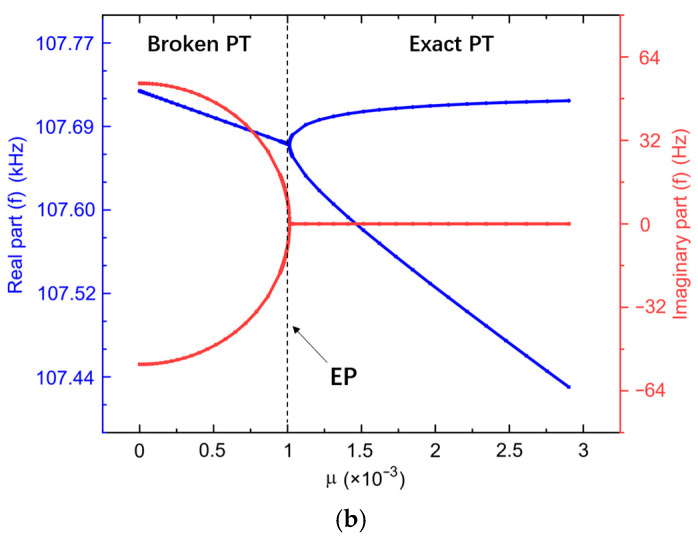
Simulation results of the PT-symmetric-coupled silicon resonators. (**a**) Keeping *μ* = 0.001, the variation of the eigenfrequencies versus *g*; (**b**) keeping *g* = 0.001, the variation of the eigenfrequencies versus *μ*.

The coupling coefficient *μ* of the coupled resonators was set to 0.001, while scanning the gain factor *g*. The variation curve of the eigenfrequencies of the PT-symmetric-coupled silicon resonators is depicted in Figure 4a. When *g* is larger than 0.001, the system is in the broken PT symmetry regime, where the real part of the eigenfrequencies merges and the imaginary part splits. When *g* is equal to 0.001, the system is at the phase-transition point EP. When *g* is less than 0.001, the system is in the exact PT symmetry regime, where the real part of the eigenfrequency splits and the imaginary part returns to zero.

The gain factor *g* of the coupled resonators was set to 0.001, while scanning the coupling coefficient *μ*. The variation curve of the eigenfrequencies of the system is shown in Figure 4b. It can be seen that when *μ* is small the system is in broken PT, and when *μ* is large, the system is in exact PT. Furthermore, as the coupling coefficient *μ* increases, the real part of the eigenfrequencies show a downward trend.

**Figure 5:**
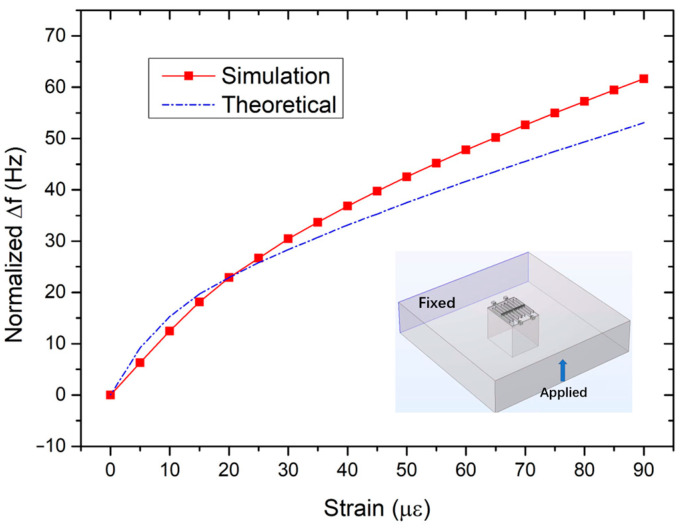
FEM simulated and theoretical calculated strain-induced frequency splitting in the PT system. The inset shows how strain is applied onto the coupled silicon resonators.

After setting the initial state of the PT system at EP, strain perturbations were applied onto the coupled silicon resonators. The simulated frequency splitting of the PT system was obtained and shown in Figure 5. For comparison, the theoretical results are also given in Figure 5. The inset in Figure 5 shows how strain was applied onto the coupled silicon resonators. As can be seen, the simulation results are consistent with the calculation results. Both results show that the PT system has a larger response slope at small applied strains, and as the applied strain increases, the response slope decreases and tends to a constant value.

### 3.3. Implementation of Negative Damping

According to PT symmetry theory, Resonator 1 requires a negative damping, which is equal in magnitude to the positive damping of Resonator 2. Here, the negative damping was implemented by a feedback loop.

When applying a feedback force *F_fb_* proportional to the velocity of Resonator 1, the equivalent damping coefficient *c_gian_* generated by *F_fb_* is:

(9) $$F_{fb}=c_{gain}\dot{x}$$

Under the influence of this feedback force, the dynamic equation of Resonator 1 becomes:

(10) $$m\ddot{x}+c\dot{x}+kx=F_{fb}$$

So, due to the feedback force, the equivalent damping coefficient *c_eff_* of Resonator 1 is calculated as:

(11) $$c_{eff}=c-c_{gain}$$

Therefore, when the damping coefficient *c_gain_* caused by the feedback force *F_fb_* is larger than the initial damping *c* of Resonator 1, it obtains an equivalent negative damping. Adjusting the feedback force applied on Resonator 1, when the negative damping *c_eff_* of Resonator 1 and the positive damping *c* of Resonator 2 satisfy *c_eff_* = −*c*, the coupled silicon resonators form PT symmetry.

The feedback force could be generated by the feedback circuit loop. The feedback force adjustment circuit is composed of a transconductance amplifier (TIA), bandpass filter (BPF), voltage-controlled amplifier (VCA), and phase shifter (PS). Firstly, the current signal of the resonator motion obtained from the V_sen_ electrode is converted into a voltage signal through TIA. Then, the voltage signal is filtered by a BPF. After that, the amplitude and phase of the voltage signal are adjusted using a VCA and PS. Finally, the feedback signal is applied back to the resonator through the V_fb_ electrode.

**Figure 6:**
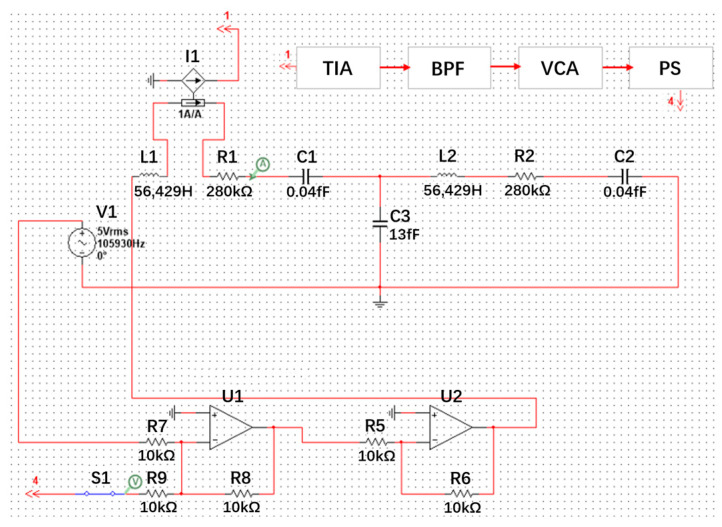
A circuit model of PT-symmetric-coupled silicon resonators for the design and verification of the feedback circuit.

A circuit model of PT-symmetric-coupled silicon resonators was established for the design and verification of the feedback circuit. The circuit model with the feedback circuit is simulated in Multisim 14, as shown in Figure 6. Among them, *R1*, *L1,* and *C1* are the equivalent circuit model of Resonator 1; *R2*, *L2,* and *C2* are the equivalent circuit model of Resonator 2; and *C3* is an equivalent capacitor representing the coupling between the two resonators. According to the mechanical parameters, the equivalent resistance, equivalent inductance, and equivalent capacitance values of a mechanical resonator can be calculated by conversion formulas [29]. Here, for verification of the feedback circuit, we set *R1* = *R2* = 28 MΩ, *C1* = *C2* = 0.04 fF, and *L1* = *L2* = 56,429 H.

**Figure 7:**
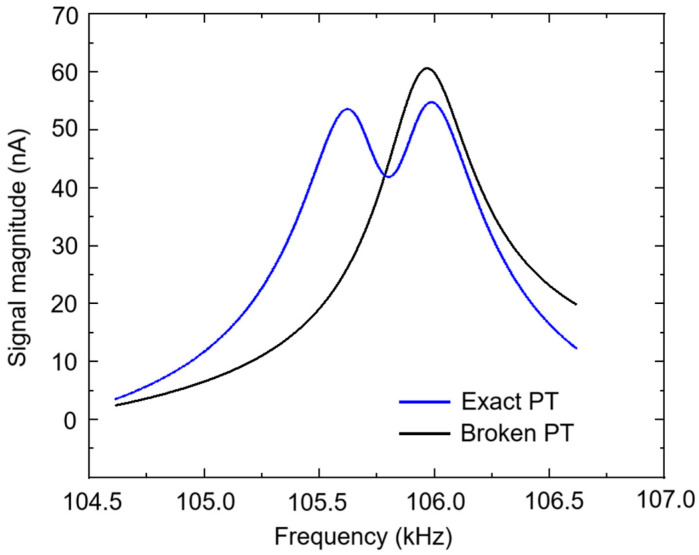
The signal waveforms of the PT system in different regimes, in which the blue line denotes the exact PT symmetry regime when *C3* = 1.3 fF; the black line denotes the broken PT symmetry regime when *C3* = 13 fF.

By adjusting the parameters of each module in the feedback loop, the gain Resonator 1 is balanced with the loss Resonator 2, *g* = *γ* = 0.01, and the system achieves PT symmetry. Then, by adjusting the coupling capacitor *C3*, the signal waveforms of the PT system in different regimes can be displayed, as shown in Figure 7. When *C3* = 1.3 fF, the equivalent coupling coefficient *μ* = 0.03, which is larger than the gain/loss coefficient of 0.01. The PT system enters the exact PT symmetry regime and displays two split resonance peaks; When *C3* = 13 fF, the equivalent coupling coefficient *μ* = 0.003, which is less than the gain/loss coefficient of 0.01. The PT system enters the broken PT symmetry regime and shows one merged resonance peak. The simulation results of the circuit model verify the effectiveness of the feedback loop. Compared to mechanical model in Section 3.2, the advantage of the circuit model here is that it is convenient for the design and simulation of the feedback loop.

## 4. Measurements and Results

### 4.1. Fabrication

**Figure 8:**
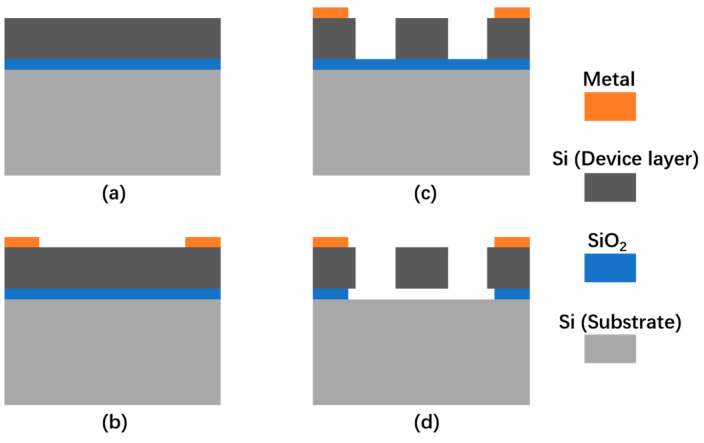
The fabrication process of the coupled silicon resonators. (**a**) A SOI wafer was prepared; (**b**) The electrodes were formed; (**c**) The structures of the resonators were formed; (**d**) The resonators were released.

The coupled silicon resonators were fabricated based on a simple SOI process. The fabrication process is depicted in Figure 8: (Figure 8a) A SOI wafer with 25 μm device layer, 2 μm SiO_2_ layer, and 400 μm substrate was prepared; (Figure 8b) then, a 50/500 nm Cr/Au layer was sputtered and patterned as the electrodes; (Figure 8c) after that, the device layer was patterned and etched by DRIE to form the structures of the resonators; (Figure 8d) finally, the SiO_2_ layer was etched by HF gas, and the resonators were released.

**Figure 9:**
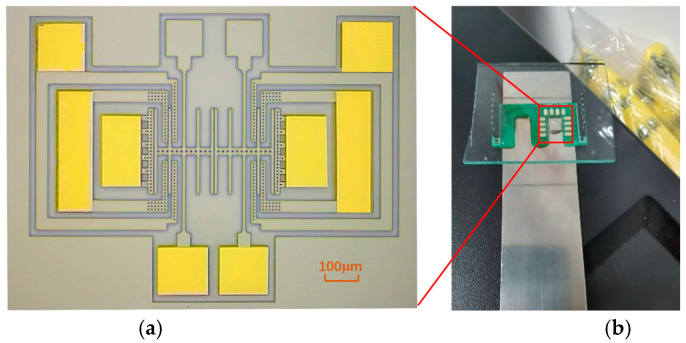
(**a**) The fabricated resonator chip. (**b**) The resonator chip adhered onto an elastic steel sheet for strain testing.

The fabricated resonator chip is shown in Figure 9a. In order to ensure symmetry, the resonant structures and electrode structures on both sides are designed to be completely identical, even if some electrodes are not used during the testing process. To conduct the strain test, the fabricated resonator chip was adhered onto a 65 Mn elastic steel sheet with a thickness of 1 mm and a length of 9 cm, as shown in Figure 9b. Then, the resonator electrodes were wire-bonded to the surrounding PCB board for testing. Here the high-temperature-resistant epoxy resin with the product model 353 ND was adopted as the adhesion layer.

### 4.2. Measurements and Results

**Figure 10:**
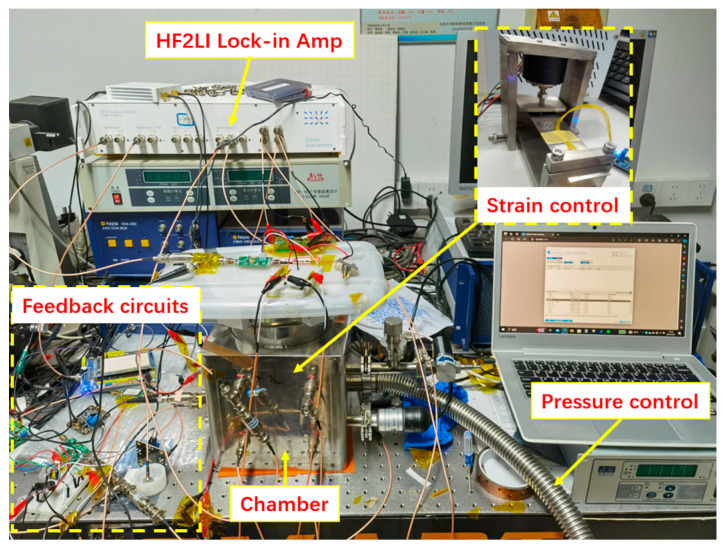
The testing platform for the PT-symmetric-coupled silicon resonators.

The testing platform for PT-symmetric-coupled silicon resonators is shown in Figure 10. The entire testing system was placed on a vibration isolation platform. In order to accurately adjust the strain of the elastic steel sheets, a servo control system was developed. The servo control system and elastic steel plate were placed in a steel chamber with adjustable air pressure. By connecting an external vacuum pump, the air pressure in the steel chamber can be adjusted between 1 and 10^5^ Pa. The feedback circuits loop included a transconductance amplifier, bandpass filter, voltage-controlled amplifier, and phase shifter. The frequency responses of the resonators were read out using a Zurich Instruments HF2LI lock-in amplifier.

Firstly, the coupled silicon resonators were adjusted to be PT symmetry. The pressure of the vacuum chamber was set to 200 Pa, at which point the loss coefficient of the loss resonator was 7 × 10^−4^. The gain and phase of the feedback circuit were carefully adjusted to make the gain coefficient of the gain resonator also 7 × 10^−4^. When the gain and loss are equal, the system is in PT symmetry.

**Figure 11:**
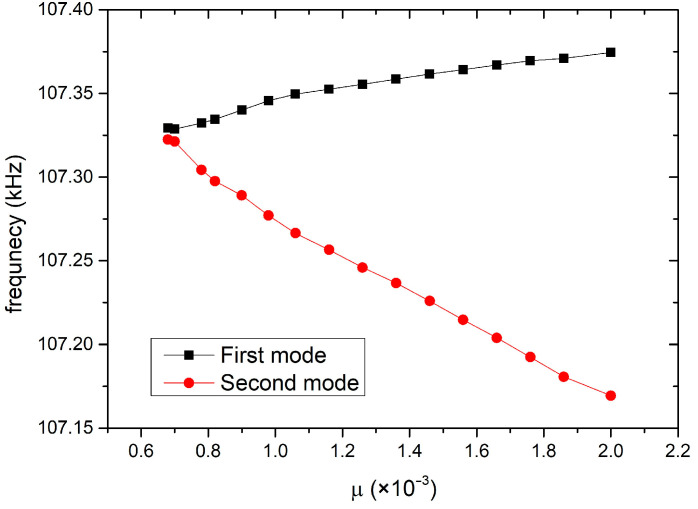
Variations of the eigenfrequencies of the PT system vs. the coupling coefficient.

Then, the coupling coefficient was scanned to find and bias the system near the EP. By adjusting the coupling voltage between two resonators, the variations of eigenfrequencies versus the coupling coefficient was obtained in Figure 11. It can be seen that when the coupling coefficient is 7 × 10^−4^, i.e., the corresponding coupling voltage is 9.8 V, the system is near the EP point.

After that, the strain-induced frequency splitting of the PT-symmetric-coupled silicon resonators was tested. The strain loading method was similar to our previous work [30], and the strain applied to the resonator chip *ε_x_* can be calculated as [31]:

(12) $$\varepsilon_{x}=\frac{3t_{c}\delta_{y}\left(L_{c}-d\right)}{2L_{c}^{3}}$$

where *L_c_* is the length of the cantilever beam, *t_c_* is its thickness, and *d* is the distance from the resonator chip to the anchor. Here, the servo motor is used to precisely control the deflection *δ_y_* of the steel plate.

**Figure 12:**
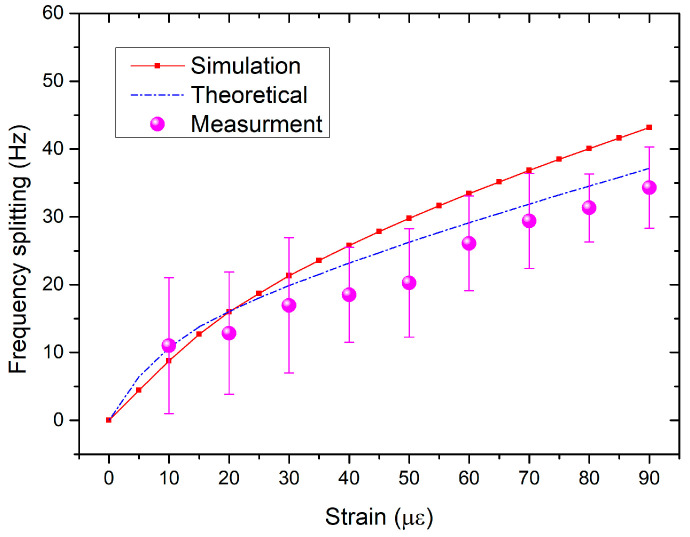
Measured strain-induced frequency splitting of the PT system. The simulation and theoretical results are also shown for comparison.

Figure 12 shows the relationship curve between frequency splitting and the applied strain of the PT system. For comparison, the simulation and the theoretical results were recalculated according to the experimental settings and are also shown in Figure 12. All curves showed that the frequency splitting increases with the increase of applied strain. The measured frequency splitting in the PT system achieved 34 Hz at 90 με. In Figure 12, the measured frequency splitting is smaller compared to the simulation or theoretical results. This may be because a portion of the applied strain is lost on the adhesive layer between the resonator chip and the steel plate [30].

## 5. Conclusions

In summary, this paper proposes the strain-induced frequency splitting in PT symmetry-coupled silicon resonators. The frequency splitting of the PT system to perturbations caused by strain was derived. The mechanical model of the PT system was established in COMSOL software and the characteristic curves of its eigenfrequencies were obtained. Theory and simulation both indicate that the PT system is more sensitive to strain perturbation near the EP point. A feedback circuit was designed to achieve the negative damping required for PT symmetry. After design and simulation, the silicon resonator chip was successfully fabricated based on an SOI process. Finally, the PT-symmetric-coupled silicon resonators were successfully constructed, and the frequency splitting phenomenon caused by strain was observed experimentally. Our work enriches the PT symmetry theory and provides valuable references for potential applications.

## Figures and Tables

**Table 1:** The key parameters of the coupled silicon resonators.

Parameter	Value
Length of the resonators	500 μm
Width of the resonators	8 μm
Thickness of the resonators	25 μm
Length of the capacitor plates	260 μm
Width of the capacitor plates	8 μm
Electrical coupling gap	2.5 μm
Young’s modulus	165 GPa
Poisson’s ratio	0.27
Density	2350 kg/m^3^

## Data Availability

The original contributions presented in the study are included in the article, further inquiries can be directed to the corresponding author.

## References

[1] Bender C.M., Boettcher S. (1998). Real Spectra in Non-Hermitian Hamiltonians Having PT Symmetry. Phys. Rev. Lett..

[2] Bender C.M. (2018). PT Symmetry: In Quantum and Classical Physics.

[3] El-Ganainy R., Makris K.G., Khajavikhan M., Musslimani Z.H., Rotter S., Christodoulides D.N. (2018). Non-Hermitian physics and PT symmetry. Nat. Phys..

[4] El-Ganainy R., Makris K.G., Christodoulides D.N., Musslimani Z.H. (2007). Theory of coupled optical PT-symmetric structures. Opt. Lett..

[5] Klaiman S., Günther U., Moiseyev N. (2008). Visualization of Branch Points in PT-Symmetric Waveguides. Phys. Rev. Lett..

[6] Schindler J., Li A., Zheng M.C., Ellis F.M., Kottos T. (2011). Experimental study of active LRC circuits with PT symmetries. Phys. Rev. A.

[7] Zhu X., Ramezani H., Shi C., Zhu J., Zhang X. (2014). PT-Symmetric Acoustics. Phys. Rev. X.

[8] Fleury R., Sounas D.L., Alù A. (2016). Parity-Time Symmetry in Acoustics: Theory, Devices, and Potential Applications. IEEE J. Sel. Top. Quantum Electron..

[9] Lin Z., Ramezani H., Eichelkraut T., Kottos T., Cao H., Christodoulides D.N. (2011). Unidirectional Invisibility Induced by PT-Symmetric Periodic Structures. Phys. Rev. Lett..

[10] Ramezani H., Christodoulides D.N., Kovanis V., Vitebskiy I., Kottos T. (2012). PT-Symmetric Talbot Effects. Phys. Rev. Lett..

[11] Alexeeva N.V., Barashenkov I.V., Sukhorukov A.A., Kivshar Y.S. (2012). Optical solitons in PT -symmetric nonlinear couplers with gain and loss. Phys. Rev. A.

[12] Hodaei H., Miri M., Heinrich M., Christodoulides D.N., Khajavikhan M. (2014). Parity-time–symmetric microring lasers. Science.

[13] Zhou H., Lee J., Liu S., Zhen B. (2019). Exceptional surfaces in PT -symmetric non-Hermitian photonic systems. Optica.

[14] Assawaworrarit S., Yu X., Fan S. (2017). Robust wireless power transfer using a nonlinear parity–time-symmetric circuit. Nature.

[15] Chen P., Sakhdari M., Hajizadegan M., Cui Q., Cheng M.M., El-Ganainy R., Alù A. (2018). Generalized parity–time symmetry condition for enhanced sensor telemetry. Nat. Electron..

[16] Zhou B., Deng W., Wang L., Dong L., Huang Q. (2020). Enhancing the Remote Distance of LC Passive Wireless Sensors by Parity-Time Symmetry Breaking. Phys. Rev. Appl..

[17] Cheng S., Li W., Zhang H., Akhtar M.N., Yi Z., Zeng Q., Ma C., Sun T., Wu P., Ahmad S. (2024). High sensitivity five band tunable metamaterial absorption device based on block like Dirac semimetals. Opt. Commun..

[18] Chen Z., Cheng S., Zhang H., Yi Z., Tang B., Chen J., Zhang J., Tang C. (2024). Ultra wideband absorption absorber based on Dirac semimetallic and graphene metamaterials. Phys. Lett. A.

[19] Charumathi P.R., Senthilnathan K. (2024). Unravelling PT Symmetry: Applications in Metamaterials. Plasmonics.

[20] Liu Z., Zhang J., Özdemir Ş.K., Peng B., Jing H., Lü X., Li C., Yang L., Nori F., Liu Y. (2016). Metrology with PT -Symmetric Cavities: Enhanced Sensitivity near the PT -Phase Transition. Phys. Rev. Lett..

[21] Zeng C., Sun Y., Li G., Li Y., Jiang H., Yang Y., Chen H. (2019). Enhanced sensitivity at high-order exceptional points in a passive wireless sensing system. Opt. Express.

[22] Hodaei H., Hassan A.U., Wittek S., Garcia-Gracia H., El-Ganainy R., Christodoulides D.N., Khajavikhan M. (2017). Enhanced sensitivity at higher-order exceptional points. Nature.

[23] Dong Z., Li Z., Yang F., Qiu C., Ho J.S. (2019). Sensitive readout of implantable microsensors using a wireless system locked to an exceptional point. Nat. Electron..

[24] Xiao Z., Li H., Kottos T., Alù A. (2019). Enhanced Sensing and Nondegraded Thermal Noise Performance Based on PT -Symmetric Electronic Circuits with a Sixth-Order Exceptional Point. Phys. Rev. Lett..

[25] Zhou B., Wang L., Dong L., Huang Q. (2021). Observation of the perturbed eigenvalues of PT-symmetric LC resonator systems. J. Phys. Commun..

[26] Zhang M., Dong L., Wang L., Huang Q. (2024). Exceptional points enhance sensing in silicon micromechanical resonators. Microsyst. Nanoeng..

[27] Saleem M.M., Saghir S., Bukhari S.A.R., Hamza A., Shakoor R.I., Bazaz S.A. (2021). A Low-g MEMS Accelerometer with High Sensitivity, Low Nonlinearity and Large Dynamic Range Based on Mode-Localization of 3-DoF Weakly Coupled Resonators. Micromachines.

[28] Wang K., Xiong X., Wang Z., Cai P., Ma L., Zou X. (2022). Utilizing the Intrinsic Mode of Weakly Coupled Resonators for Temperature Compensation. Micromachines.

[29] Lee J.E. (2008). Silicon Micromechanical Resonators for Measurements of Mass and Charge. Ph.D. Thesis.

[30] Zhang C., Zhang S., Wang L. (2021). A Sawtooth MEMS Capacitive Strain Sensor for Passive Telemetry in Bearings. IEEE Sens. J..

[31] Suster M., Guo J., Chaimanonart N., Ko W.H., Young D.J. Low-noise CMOS integrated sensing electronics for capacitive MEMS strain sensors. Proceedings of the IEEE 2004 Custom Integrated Circuits Conference.

